# Fatigue After Aneurysmal Subarachnoid Hemorrhage: Clinical Characteristics and Associated Factors in Patients With Good Outcome

**DOI:** 10.3389/fnbeh.2021.633616

**Published:** 2021-05-12

**Authors:** Elin Western, Tonje Haug Nordenmark, Wilhelm Sorteberg, Tanja Karic, Angelika Sorteberg

**Affiliations:** ^1^Department of Neurosurgery, Division of Clinical Neuroscience, Oslo University Hospital, Oslo, Norway; ^2^Department of Physical Medicine and Rehabilitation, Division of Clinical Neuroscience, Oslo University Hospital, Oslo, Norway; ^3^Department of Psychology, Faculty of Social Sciences, University of Oslo, Oslo, Norway; ^4^Faculty of Medicine, Institute of Clinical Medicine, University of Oslo, Oslo, Norway

**Keywords:** aneurysmal subarachnoid hemorrhage (aSAH), fatigue, mood disorders, cognitive function, health-related quality of life (HRQoL), return to work (RTW)

## Abstract

Fatigue after aneurysmal subarachnoid hemorrhage (post-aSAH fatigue) is a frequent, often long-lasting, but still poorly studied sequel. The aim of the present study was to characterize the nature of post-aSAH fatigue with an itemized analysis of the Fatigue Severity Scale (FSS) and Mental Fatigue Scale (MFS). We further wanted to assess the association of fatigue with other commonly observed problems after aSAH: mood disorders, cognitive problems, health-related quality of life (HRQoL), weight gain, and return to work (RTW). Ninety-six good outcome aSAH patients with fatigue completed questionnaires measuring fatigue, depression, anxiety, and HRQoL. All patients underwent a physical and neurological examination. Cognitive functioning was assessed with a neuropsychological test battery. We also registered prior history of fatigue and mood disorders as well as occupational status and RTW. The patients experienced fatigue as being among their three most disabling symptoms and when characterizing their fatigue they emphasized the questionnaire items “low motivation,” “mental fatigue,” and “sensitivity to stress.” Fatigue due to exercise was their least bothersome aspect of fatigue and weight gain was associated with depressive symptoms rather than the severity of fatigue. Although there was a strong association between fatigue and mood disorders, especially for depression, the overlap was incomplete. Post-aSAH fatigue related to reduced HRQoL. RTW was remarkably low with only 10.3% of patients returning to their previous workload. Fatigue was not related to cognitive functioning or neurological status. Although there was a strong association between fatigue and depression, the incomplete overlap supports the notion of these two being distinct constructs. Moreover, post-aSAH fatigue can exist without significant neurological or cognitive impairments, but is related to reduced HRQoL and contributes to the low rate of RTW.

## Introduction

Aneurysmal subarachnoid hemorrhage (aSAH) is a devastating disease with case fatality rates of 27–44% (Nieuwkamp et al., 2009). Even though the mortality rates are high, survival has improved due to early intervention and advances in management. Remarkably, most aSAH survivors recover without significant neurological deficits, however, even patients with good outcome report substantial problems with fatigue, as well as cognitive and emotional problems (Al-Khindi et al., 2010; Nordenmark et al., 2019a; Nussbaum et al., 2020; Tang et al., 2020). Fatigue represents the most frequent symptom after aSAH and is found in 50–70% of patients even several years after the hemorrhage (Kutlubaev et al., 2012; Western et al., 2020).

There is no consensus concerning a definition of fatigue. Chaudhuri and Behan (2004) have described fatigue as problems with the initiation of or sustaining voluntary activities. They discriminate between peripheral fatigue at the muscular level and fatigue originating in the central nervous system (central fatigue). The latter is characterized as a feeling of constant exhaustion and may have a cognitive component (mental fatigue). Mental fatigue is a prominent symptom in a diversity of neurological diseases (Chaudhuri and Behan, 2004; Yoshii et al., 2006; Penner and Paul, 2017; Arm et al., 2019). Fatigue after aSAH has been less extensively investigated. Buunk et al. (2018) found mental fatigue to prevail in aSAH patients. Levels of fatigue after aSAH were also found to be related to outcome measures like Return to Work (RTW) and Glasgow outcome score extended (GOSE). In the study by Sörbo et al. (2019), a majority (57%) of aSAH patients experienced mental fatigue, however a more precise delineation of the core problems in individuals with post-aSAH fatigue is still lacking.

Not only the definition, but also the quantification of fatigue is challenging, and currently, questionnaires are the only validated tools available to this end (De Doncker et al., 2018). One of the most commonly used questionnaires is the Fatigue Severity Scale (FSS; Krupp et al., 1989) assessing the impact of fatigue on daily life. A relatively new scale, the Mental Fatigue Scale (MFS; Johansson et al., 2010), has been developed for the assessment of mental fatigue and related symptoms and could possibly give more information regarding the mental aspects of fatigue than the FSS. Combining the information collected by the FSS and MFS could hence provide further characterization of the nature of fatigue after aSAH. In clinical experience, many aSAH patients also complain about a long–term weight gain that they cannot explain by nutritional changes. Inactivity may contribute to this weight gain; inactivity possibly caused by fatigue. When presently investigating fatigue, we, therefore, included weight change over time to see if weight gain represents a surrogate marker for the severity of physical fatigue.

In addition to fatigue, mood disorders, and cognitive problems, aSAH survivors commonly experience reduced Health-Related Quality of Life (HRQoL) and a low rate of RTW (Visser-Meily et al., 2009; Passier et al., 2011b; Czapiga et al., 2013; Harris, 2014; Taufique et al., 2016; Buunk et al., 2019). A more precise knowledge of the challenges fatigue poses could facilitate targeted rehabilitation programs. Moreover, it is not clear to which extent fatigue contributes to mood disorders and cognitive problems. In this respect, it is relevant to know if mood disorders are secondary to or amplified by fatigue rather than representing an autonomous entity requiring antidepressant or anxiolytic treatment.

The aim of the present study was to characterize the nature of fatigue after aSAH with an itemized analysis of the FSS and MFS questionnaires. We further wanted to assess if and how fatigue after aSAH associates with other commonly observed problems after aSAH like mood disorders, cognitive problems, reduced HRQoL, weight gain, and RTW. To this end, we investigated 96 patients in the chronic phase after aSAH that all suffered from fatigue and had been included in a clinical trial to investigate the effect of the substance (-)-OSU6162 on fatigue after aSAH.

## Materials and Methods

Data for the present study were acquired within the randomized clinical trial (RCT) “OSU6162 in the treatment of fatigue and other hage—a double-blind, randomized, placebo-controlled study” (EudraCT no. 2016-004739-19; ClinicalTrials.gov Identifier: NCT03209830). The study was also approved by the regional ethics committee (REC, reference: 2016/2214). The substance (-)-OSU6162 is a stabilizer of the neurotransmitter dopamine and studies have shown that it may have a positive effect on fatigue (Johansson et al., 2012; Kloberg et al., 2014; Haghighi et al., 2018; Nilsson et al., 2018).

### Patients

Patients (≥18 years) in the chronic phase (≥1 year) after treatment at our hospital for aSAH between January 2012 and March 2018, and permanently living in the South-Eastern Norway Regional Health Authority were eligible. A clinical neuropsychologist conducted the recruitment by telephone interview. All patients with a mean FSS score ≥4 were invited for assessment at the hospital. We excluded patients unable to conduct the different assessments, handle the instruments used for evaluation, or those who were unable to give consent. We also excluded those that had undergone brain surgery <12 months prior to inclusion, patients with brain tumor, cerebral arteriovenous malformation, inadequately treated hydrocephalus, epilepsy, cerebral paresis, and neurodegenerative disease. Pregnancy also prohibited participation, as did active drug abuse, severe blood test deviations from normal and an electrocardiogram with prolonged QTc time >480 ms.

Due to possible interactions with (-)-OSU6162, we further excluded patients using metabolic enzyme inhibitors and inducers and those using drugs with a narrow therapeutic window or requiring individual dose adjustments. The following Anatomical Therapeutic Chemical (ATC) Classification System categories were not allowed: N06B A+X, N07A, N06A G+X, N05A, N03A, J04A, J01D H, L04A D, B01A A, C01A A, H01B A, and N04B D.

### Assessments

#### Demographic Data/aSAH Characteristics

Age, gender, weight, height, education level, and pre-ictal/current work status was recorded during the baseline visits of the RCT. During the same visits, the patients also underwent a neurological examination and were later scored using the National Institutes of Health Stroke Scale (NIHSS). Weight at ictus was retrieved from the medical journals. Change in body mass index (BMI, kg/m^2^) from ictus to baseline assessment in the study was calculated and adjusted for months since the ictus. From the medical journals, we also retrieved the clinical condition according to Hunt and Hess (Hunt and Hess, 1968) just prior to aneurysm repair or prior to intubation in those admitted intubated, aneurysm localization, method of aneurysm repair, and any treatment for acute hydrocephalus (need of external drainage of cerebrospinal fluid) or chronic hydrocephalus (implanted shunt). We noted the acquisition of any radiologically documented new cerebral infarction during the acute phase of aSAH, regardless of cause. Clinical outcome at one year after the ictus was assessed using the modified Rankin Score (Rankin, 1957; van Swieten et al., 1988).

#### Fatigue

A prior history of fatigue was registered from the medical records at aSAH admission and by asking the patient: “did you experience fatigue before your aSAH” (yes/no). Patients who answered “yes” were encouraged to describe, regardless of cause, the nature of their pre-ictal fatigue. Those who reported pre-ictal fatigue with an intensity and severity that caused problems in their daily lives were defined as having fatigue before the hemorrhage (Lynch et al., 2007).

Post-aSAH fatigue was measured using the Norwegian version of the FSS (Krupp et al., 1989; Lerdal et al., 2005) and the MFS (Johansson et al., 2010). FSS consists of nine items about the impact of fatigue on daily life, where each statement is scored on a 7-point Likert scale ranging from 1 (strongly disagree) to 7 (strongly agree). The nine items of the FSS questionnaire are: (1) My motivation is lower when I am fatigued; (2) Exercise brings on my fatigue; (3) I am easily fatigued; (4) Fatigue interferes with my physical functioning; (5) Fatigue causes frequent problems for me; (6) My fatigue prevents sustained physical functioning; (7) Fatigue interferes with carrying out certain duties and responsibilities; (8) Fatigue is among my three most disabling symptoms; and (9) Fatigue interferes with my work, family, or social life. The FSS score is the mean of the nine item scores. A mean FSS score of ≥4 is considered indicative of fatigue (Krupp et al., 1989). Correspondingly, the MFS questionnaire comprises 15 questions regarding affective, cognitive, and sensory symptoms related to fatigue. The 15 items of the MFS questionnaire are: (1) Fatigue in general; (2) Lack of initiative; (3) Mental fatigue; (4) Mental recovery; (5) Concentration difficulties; (6) Memory problems; (7) Slowness of thinking; (8) Sensitivity to stress; (9) Emotional instability; (10) Irritability; (11) Sensitivity to light; (12) Sensitivity to noise; (13) Decreased sleep; (14) Increased sleep; and (15) 24-h variations. Items 1–14 are scored on a scale ranging from 0 to 3; where 0 corresponds to normal function, 1 indicates a problem, 2 indicates a pronounced problem and 3 a maximal problem. The patient can also choose an answer in between the exemplified alternatives; i.e., 0.5, 1.5, and 2.5. Item 15, which is not included in the total sum score, indicates which time of the day is felt best and worst if there is a diurnal variation. An MFS sum score of ≥10.5 is suggestive of mental fatigue (Johansson and Ronnback, 2014).

#### Mood Disorders

A prior history of depression and anxiety was registered from the medical records at aSAH admission and by asking the patient: “did you experience psychiatric problems before your aSAH” (yes/no). Patients who answered “yes” were encouraged to describe the nature of the symptoms, severity and, duration. Those who reported depressive or anxiety symptoms, often in combination with therapy or pharmacological treatment, interfering with activities of daily life, were defined as having depression or anxiety before the hemorrhage.

The Beck Depression Inventory-II (BDI-II; Beck et al., 1996) and The Beck Anxiety Inventory (BAI; Beck et al., 1988) were used to assess the frequency and severity of depressive and anxiety symptoms, respectively, during the past 2 weeks. Both scales are 21-items self-report questionnaires with each item rated on a 4-point scale from 0 to 3 with higher scores indicating more severe symptomology. A BDI-II score of ≥20 was defined as clinical depression (moderate to severe depressive symptoms) whereas a BAI score of ≥16 was defined as clinical anxiety (moderate to severe anxiety symptoms). In this study, mood disorder was defined as the presence of clinical depression and/or clinical anxiety.

#### Cognitive Function

The following six cognitive domains were examined: sensomotor function, attention, psychomotor speed, verbal learning, verbal memory, and executive function.

All patients underwent a standardized neuropsychological test battery that covered the six cognitive domains mentioned above. We selected tests that are widely used in routine neuropsychological practice and sensitive to deficits after brain injury. All tests were administered in the same order to all patients. The *Sensomotor function* was measured with Grooved Pegboard (Halstead-Reitan Neuropsychological Battery; Heaton et al., 1991) and Trail Making Test condition 5 from Delis-Kaplan Executive Function System (D-KEFS; Delis et al., 2001) whereas *Attention* was assessed using the Trail Making Test condition 1 from D-KEFS, Digit Span from Wechsler Adult Intelligence Scale—fourth edition (WAIS-IV; Wechsler, 2008), and Conners’ Continuous Performance—3rd edition (CPT-III; Conners, 2014). *Psychomotor speed* was examined using the Trail Making Test condition 2 and 3 (D-KEFS) and the Color-Word Interference Test condition 1 and 2 (D-KEFS). *Verbal learning* and *Verbal memory* were measured using the California Verbal Learning Test—Second edition (CLVT-II; Delis et al., 2000) while *Executive function* was assessed using the Trail Making Test condition 4 (D-KEFS) and Color-Word Interference Test condition 3 and 4 (D-KEFS).

All tests were scored using published norms and, where available, age-adjusted scores for a normal population. In order to compare the results with a normal population with similar demographic features, scores were converted into *z*-scores. *Z*-scores of patients on all individual tests were stratified into 4 categories: “normal” (*z*-scores > −1.00), “mild impairment” (*z*-scores between −1.00 and −1.49), “moderate impairment” (*z*-scores between −1.50 and −2.00), and “deficit” (*z*-scores <−2.00). Furthermore, the proportions of patients within these four categories were averaged per cognitive domain.

#### Health-Related Quality of Life

HRQoL was measured using the Short Form Health Survey (SF-36; Ware and Sherbourne, 1992). The SF-36 is a 36-item self-report questionnaire measuring subjective HRQoL in 8 health-related domains: physical functioning, role limitations due to physical problems (role-physical), bodily pain, general health, vitality, social functioning, role limitations due to emotional problems (role-emotional), and mental health. Scores were converted to updated norms based on the Norwegian version of SF-36 (Garratt and Stavem, 2017) and *t*-scores <37 were defined as outside the normal range.

#### Return to Work

Only patients employed at the time of hemorrhage were included in the RTW analysis. The patients were scored as follows: no RTW (work before but not after aSAH), partial RTW (reduced work after as compared to before aSAH), and full RTW (same amount of work before and after aSAH).

### Statistical Analysis

Statistical analysis was performed with IBM SPSS version 25 for Windows (Armonk, NY: IBM Corp). Continuous variables were presented by mean and standard deviation, and independent samples *t*-test was used to compare differences between groups. Continuous variables which were not normally distributed were presented with median and range, and the Mann–Whitney *U* test was used for differences between groups. Categorical variables were presented as frequencies or percentages, and we used the Chi-Square test to compare differences between groups. The itemized analysis was conducted by comparing the mean FSS and mean MFS item scores against the mean FSS score ± 2 SEM and mean MFS item score ± 2 SEM for the whole group (*N* = 96). FSS and MFS item scores defined as prominent features of post-aSAH fatigue also corresponded to results of skewness (i.e., highest negative values). Bivariate and partial Pearson correlation coefficient was used to explore FSS and MFS with continuous variables and adjust for covariates. Statistical significance was set at 0.05 (two-sided).

## Results

### Patients

**Figure 1:**
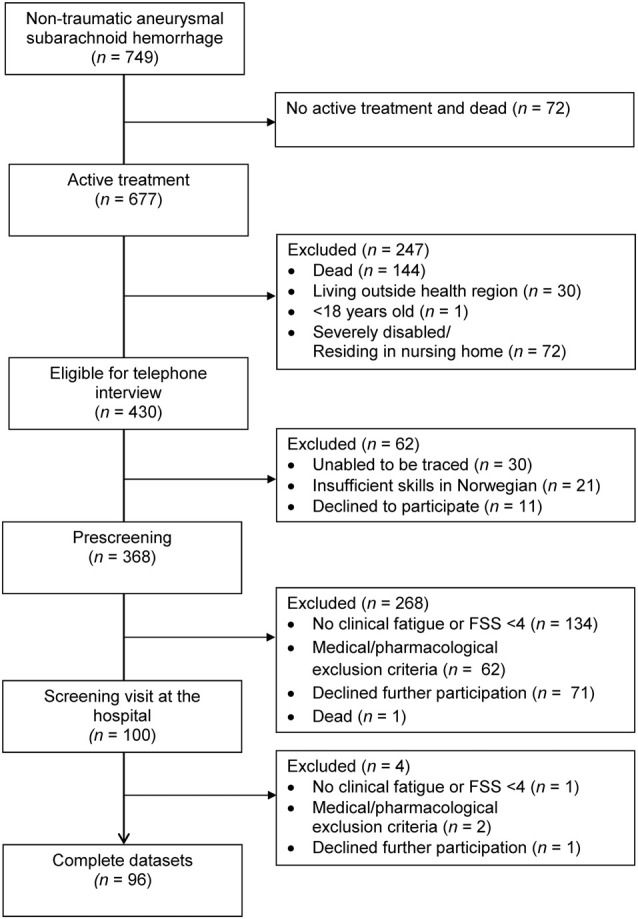
Flow chart of patient enrollment.

A total of 749 patients were admitted for aSAH between January 2012 and March 2018, of whom 677 received active treatment, 430 patients were eligible for a telephone interview, 368 completed prescreening by telephone and 96 patients completed the full assessment at the hospital (see Figure 1). The time from hemorrhage to inclusion was median 25 months (range 12–83 months). The characteristics of the 96 included patients are displayed in Table 1. Whereas the 96 included and the 272 excluded patients did not differ with respect to gender [$X_{\left(1\right)}^{2}$ = 0.02, *p* = 0.886], aneurysm location [$X_{\left(1\right)}^{2}$ = 0.38, *p* = 0.540] or mode of aneurysm repair [$X_{\left(1\right)}^{2}$ = <0.01, *p* = 0.969], the excluded patients were significantly older at the time of hemorrhage [*t*_(1)_ = 3.58, *p* = <0.001] and in better clinical condition (HH 1–3 vs. HH 4–5) prior to aneurysm repair as compared to the included patients [$X_{\left(1\right)}^{2}$ = 4.29, *p* = 0.038].

**Table 1:** Characteristics of patients with post-aSAH fatigue (*N* = 96).

	*n*	%
Demographic characteristics
Age (years) at assessment, median (range)	57 (22–74)
Sex, female	65	67.7
Body Mass Index (BMI)
BMI at ictus, mean ± SD (range)	26.8 ± 5.4 (15.8–41.2)
Education level
Lower secondary school	13	13.5
Upper econdary school	36	37.5
Undergraduate school	33	34.4
Graduate school	14	14.6
Work status at ictus
Paid work	78	81.3
Retirement/disability leave	15	15.6
Student	3	3.1
Prior history of fatigue and mood disorders
Fatigue before aSAH	13	13.5
Depression before aSAH	22	22.9
Anxiety before aSAH	12	12.5
aSAH characteristics
Time since ictus (months), median (range)	25 (12–83)	
Method of aneurysm repair
Surgical	43	44.8
Endovascular	53	55.2
Aneurysm localization
Anterior circulation	83	86.5
Posterior circulation	13	13.5
Hunt and Hess (HH)
1	25	26.0
2	33	34.4
3	12	12.5
4	16	16.7
5	10	10.4
Acute hydrocephalus	69	71.9
Chronic hydrocephalus	24	25.0
Cerebral infarction*	31	32.3
Characteristics post-aSAH
Outcome (modified Rankin Score)
0	5	5.2
1	68	70.8
2	23	24.0
Neurological status at assessment
NIHHS score, mean ± SD (range)	0.7 ± 1.0 (0–5)	
Body Mass Index (BMI)
BMI at assessment, mean ± SD	29.3 ± 6.5 (16.5–56.8)	
(range)
Monthly change in BMI,	0.1 ± 0.2 (−0.7–0.8)	
mean ± SD (range)

Data are presented as the absolute number of patients with percentages in parentheses with the exception of BMI and NIHHS score, which is listed as mean ± standard deviation and range. Age and time since ictus is reported as median and range. *All new cerebral infarction registered independent of cause (i.e., primary or secondary).

### Post-aSAH Fatigue

For all 96 patients, the mean FSS score was 6.0 ± 0.8, and the MFS sum score was 18.1 ± 5.6. Scores on the two fatigue measures were closely connected, where higher scores on FSS were significantly related to higher scores on MFS [*r*_(94)_ = 0.47, *p* = <0.001].

**Figure 2:**
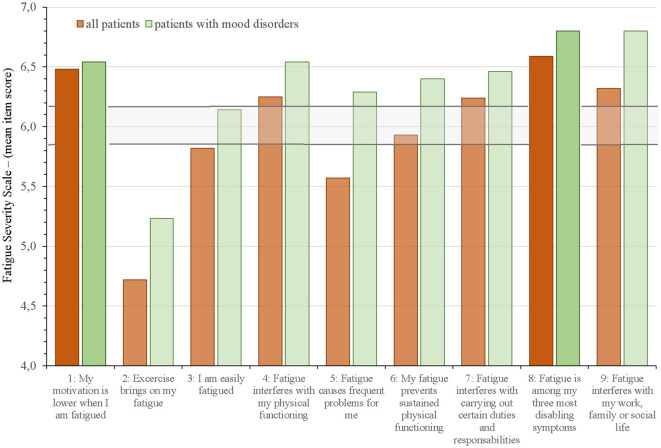
Mean Fatigue Severity Scale (FSS) item scores for all aSAH patients and allocation into subgroup of patients with mood disorders against the mean FSS score ± 2 SEM (horizontal lines). The two FFS items with the highest mean scores are highlighted.

**Figure 3:**
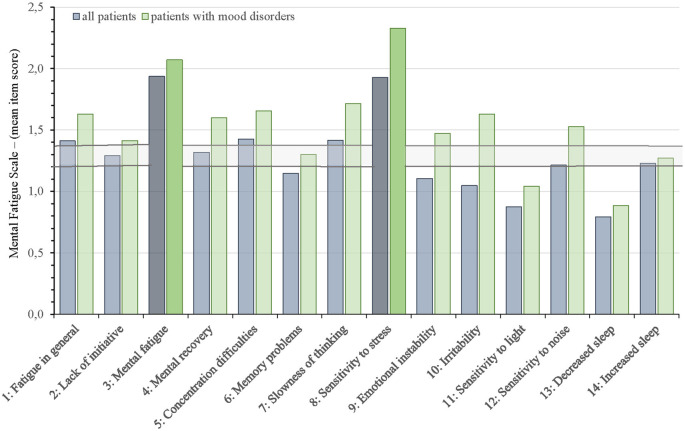
Mean Mental Fatigue Scale (MFS) item scores for all aSAH patients and allocation into subgroup of patients with mood disorders against the mean MFS item score ± 2 SEM (horizontal lines). The two MFS items with the highest mean scores are highlighted.

Figures 2 and 3 show which of the nine FSS items and the 14 MFS items that stood out as most and less prominent by displaying the mean scores for each item for the entire group and those with mood disorders against the mean FFS score ± 2 SEM and the mean MFS item score ± 2 SEM.

On the FSS questionnaire (Figure 2), the two items that were scored highest by the entire group was “My motivation is lower when I am fatigued” (item 1: 6.5 ± 1.0) and “Fatigue is among my three most disabling symptoms” (item 8: 6.6 ± 0.7). The items “Exercise brings on my fatigue” (item 2: 4.7 *±* 2.0) and “Fatigue causes frequent problems for me” (item 5: 5.6 ± 1.4) had the lowest scores; however, within these two items we found the largest differences between all patients and the subgroup with mood disorders. Item 3 “I am easily fatigued” fell just below the ± 2SEM of mean FSS for the entire group, but scored within this range in those with mood disorders. All other items on FFS were above or within the range of cut-off (± 2 SEM).

On the MFS questionnaire (Figure 3), the items “Mental fatigue” (item 3: 1.9 *±* 0.5) and “Sensitivity to stress” (item 8: 1.9 *±* 0.9) stood out with the highest scores. Item 11 “sensitivity to light” (0.9 ± 0.7) and item 13 “decreased sleep” (0.8 ± 0.8) scored lowest. Item 6 “Memory problems”, item 9 “Emotional instability”, and item 10 ”Irritability” scored below the range of cut-off (± 2 SEM) for all 96 patients but scored within or above the range of cut-off in the subgroup of patients with mood disorders. All other items on MFS were above or within the range of cut-off (± 2 SEM).

### Fatigue and Mood Disorders

The proportion of patients within the different standardized categories for BDI-II and BAI are listed in Table 2. Clinical depression and clinical anxiety were reported by 34.4% and 18.8%, respectively.

**Table 2:** Mean score and percentage of patients with responses in the clinical range on self-assessment questionnaires.

		*n*	*M*	Range	% in clinical range*
Fatigue Fatigue Severity Scale (FSS), mean score		96	6.0 ± 0.8	3.8–7.0	99.0
Mental Fatigue Scale (MFS), sum score		96	18.1 ± 5.6	5.0–41.0	93.8
Depressive symptoms
Beck Depression Inventory-II (BDI-II), score		96	16.2 ± 8.8	1–45	34.4
	Minimal (0–13)	42			43.8
	Mild (14–19)	21			21.9
Clinical depression	Moderate (20–28)	22			22.9
	Severe (29–63)	11			11.5
Anxiety symptoms	
Beck Anxiety Inventory (BAI), score		96	8.9 ± 7.1	0–29	18.8
	Minimal (0–7)	50			52.1
	Mild (8–15)	28			29.2
Clinical anxiety	Moderate (16–25)	15			15.6
	Severe (26–63)	3			3.1
Health-Related Quality of Life (HRQoL)
Short Form Health Survey (SF-36), *t*-score				
Physical functioning		96	43.1 ± 10.8	6.0–58.5	26.0
Role-physical		96	39.8 ± 10.0	22.9–62.2	50.0
Bodily pain		96	45.1 ± 10.5	21.7–63.1	19.8
General health		96	40.5 ± 5.8	25.1–54.1	25.0
Vitality		96	35.9 ± 9.0	17.2–55.3	46.9
Social functioning		96	36.3 ± 9.3	8.0–49.2	47.9
Role-emotional		96	43.3 ± 13.7	14.2–59.0	33.3
Mental health		96	43.0 ± 12.8	13.9–64.3	29.2

*Cut-off values for clinical relevance as follows: FSS ≥4; MFS ≥10.5; BDI-II ≥20; BAI ≥16; SF-36 *t*-score <37.

There was a positive correlation between mean FSS scores and depressive symptoms (BDI-II) scores with and without adjustment for anxiety symptoms (BAI) score [*r*_(94)_ = 0.42, *p =* <0.001; *r*_adj.(93)_ = 0.34, *p* = <0.001]. In contrast, the relationship between mean FSS scores and anxiety symptoms (BAI) scores disappeared when adjusted for depressive symptoms (BDI-II) [*r*_(94)_ = 0.27, *p* = 0.007; *r*_adj.(93)_ =−0.02, *p* = 0.833]. A positive correlation was found between MFS sum scores and depressive symptoms (BDI-II) scores, also when adjusted for anxiety [*r*_(94)_ = 0.52, *p* = <0.001; *r*_adj.(93)_ = 0.30, *p* = 0.003]. Although still reaching significance, the correlation between MFS sum scores and anxiety symptoms (BAI) was weakened when adjusted for depressive symptoms (BDI-II) [*r*_(94)_ = 0.49, *p* = <0.001; *r*_adj.(93)_ = 0.21, *p* = 0.043].

Symptoms of depression and anxiety (BDI-II and BAI scores) within demographics, prior history, and aSAH characteristics are presented in Table 3. Patients with prior history of depression had both higher BDI-II scores (depressive symptoms) and higher BAI scores (anxiety symptoms) than those without a prior history of depression. In contrast, patients with prior history of anxiety had higher BAI scores (anxiety symptoms), but not higher BDI-II scores (depressive symptoms) than those with no prior history of anxiety. Except for a trend towards higher BDI-II scores (depressive symptoms) among patients with endovascular as opposed to surgical aneurysm repair (17.8 + 8.6 vs. 14.4 + 8.7, *p* = 0.058), demographical data and aSAH characteristics were not related to BDI-II and BAI scores. Mood disorders (depression and anxiety) were more common in patients undergoing endovascular than surgical aneurysm repair [47.2% vs. 23.3%, *p* = 0.019].

**Table 3:** Relationship between symptoms of depression and anxiety (BDI-II and BAI scores), demographics, prior history of fatigue and mood disorders, and hemorrhage characteristics.

	*n*	BDI-II score (M)	*t*_(95% CI)_	*p*	BAI score (M)	*t*_(95% CI)_	*p*
**Demographic data**
Age
≤57 (median)	45	15.8 *±* 7.8	−0.449 (−4.380–2.765)	0.654	9.1 ± 6.6	0.291 (−2.458–3.303)	0.772
>57	51	16.6 ± 9.6			8.7 ± 7.5
Sex
Female	65	15.6 ± 8.4	−1.070 (−5.837–1.750)	0.288	8.8 ± 7.2	−0.129 (−3.275–2.785)	0.898
Male	31	17.6 ± 9.4			9.0 ± 6.9
Education level
Medium	49	17.2 ± 9.5	1.138 (−1.513–5.579)	0.258	9.7 ± 8.3	1.247 (−1.058–4.612)	0.216
High	47	15.2 ± 7.9			8.0 ± 5.5
**Prior history**
Fatigue before aSAH
No	83	15.7 ± 8.5	−1.509 (−9.070–1.237)	0.135	8.5 ± 7.0	−1.391 (−7.075–1.245)	0.167
Yes	13	19.6 ± 9.8			11.4 ± 7.1
Depression before aSAH
No	74	14.7 ± 8.2	−3.192 (−10.517–2.451)	0.002	7.2 ± 6.6	−4.625 (−10.281–4.105)	≤0.001

Yes	22	21.2 ± 9.1			14.4 ± 5.7
Anxiety before aSAH
No	85	15.8 ± 8.6	−1.265 (−9.096–2.016)	0.209	8.1 ± 6.7	−3.001 (−10.831–2.206)	0.003
Yes	11	19.4 ± 9.9			14.6 ± 7.6
**aSAH characteristics**
Hunt and Hess (HH)
Good grade, HH 1–3	70	16.0 ± 9.2	−0.497 (−5.015–3.006)	0.620	9.0 ± 7.6	0.467 (−2.162–3.479)	0.642
Poor grade, HH 4–5	26	17.0 ± 7.6			8.4 ± 5.5
Aneurysm localization
Anterior circulation	83	16.8 ± 9.0	1.578 (−1.057–9.239)	0.118	9.2 ± 7.1	1.282 (−1.476–6.857)	0.203
Posterior circulation	13	12.7 ± 6.1			6.5 ± 6.9
Aneurysm repair
Surgery	43	14.4 ± 8.7	−1.921 (−6.926–0.115)	0.058	8.4 ± 7.4	−0.556 (−3.695–2.079)	0.580
Coiling	53	17.8 ± 8.6			9.2 ± 6.8
Acute Hydrocephalus
No	27	15.7 ± 8.9	−0.392 (−4.749–3.184)	0.696	10.3 ± 7.9	1.280 (−1.127–5.214)	0.204
Yes	69	16.5 ± 8.8			8.3 ± 6.7
Chronic hydrocephalus
No	72	17.0 ± 9.2	1.420 (−1.161–6.995)	0.159	9.6 ± 7.3	1.677 (−0.508–6.036)	0.097
Yes	24	14.0 ± 7.2			6.8 ± 5.8
Cerebral Infarction
No	65	16.5 ± 9.5	0.474 (−2.902–4.722)	0.637	9.4 ± 7.6	1.119 (−1.218–4.343)	0.267
Yes	31	15.6 ± 7.2			7.8 ± 5.7

*Independent samples t-tests comparisons between groups. Higher BDI-II and BAI scores indicate more symptoms of depression and anxiety, respectively*.

### Fatigue and Demographic Data/aSAH Characteristics

Fatigue scores vs. demographics, prior history, and aSAH characteristics are presented in Table 4. Patients with low/intermediate education level had higher mean FSS scores than those with high education level (6.3 ± 0.7 vs. 5.7 ± 0.8, *p* = 0.001) and patients with endovascular aneurysm repair had higher mean FSS scores than those with surgical aneurysm repair (6.2 ± 0.7 vs. 5.7 ± 0.8, *p* = 0.002). There were no significant relationships between MFS sum scores and any of the background variables.

**Table 4:** Relationship between mean Fatigue Severity Scale (FSS) scores, Mental Fatigue Scale (MFS) sum scores, demographics, prior history of fatigue and mood disorders, and hemorrhage characteristics.

	*n*	Mean FSS score (*M*)	*t* (95% CI)	*p*	MFS sum score (*M*)	*t* (95% CI)	*p*
**Demographic data**
Age
≤57 (median)	45	5.9 ± 0.7	−0.626 (−0.421–0.219)	0.533	18.5 ± 5.8	0.526 (−1.673–2.878)	0.600
>57	51	6.0 ± 0.8			17.9 ± 5.4
Sex
Female	65	5.9 ± 0.8	−0.902 (−0.496–0.186)	0.370	18.5 ± 5.5	0.828 (−1.413–3.433)	0.410
Male	31	6.1 ± 0.7			17.5 ± 5.7
Education level
Low/Intermediate	49	6.3 ± 0.7	3.393 (0.214–0.819)	0.001	18.9 ± 6.3	1.354 (−0.716–3.789)	0.176
High	47	5.7 ± 0.8			17.4 ± 4.6
**Prior history**							
Fatigue before aSAH
No	83	6.0 ± 0.8	−0.502 (−0.586–0.349)	0.617	18.2 ± 5.6	0.333 (−2.764–3.878)	0.740
Yes	13	6.1 ± 0.9			17.7 ± 5.8
Depression before aSAH
No	74	5.9 ± 0.8	−1.436 (−0.650–0.104)	0.154	17.6 ± 5.7	−1.760 (−5.022–0.302)	0.082
Yes	22	6.2 ± 0.8			20.0 ± 4.7
Anxiety before aSAH
No	85	6.0 ± 0.8	−1.171 (−0.794–0.205)	0.245	17.9 ± 5.5	−1.093 (−5.499–1.595)	0.277
Yes	11	6.3 ± 0.6			19.9 ± 6.5
**aSAH characteristics**
Hunt and Hess (HH)
Good grade, HH 1–3	70	6.0 ± 0.8	0.359 (−0.295–0.425)	0.721	17.9 ± 5.3	−0.573 (−3.291–1.817)	0.568
Poor grade, HH 4–5	26	5.9 ± 0.9			18.7 ± 6.4
Aneurysm localization Anterior circulation	83	6.0 ± 0.8	−0.713 (−0.634–0.299)	0.478	18.1 ± 4.9	−0.183 (−5.964–5.032)	0.858
Posterior circulation	13	6.1 ± 0.7			18.5 ± 9.0
Aneurysm repair
Surgery	43	5.7 ± 0.8	−3.218 (−0.801–0.190)	0.002	17.6 ± 5.6	−0.856 (−3.260–1.295)	0.394
Coiling	53	6.2 ± 0.7			18.6 ± 5.6
Acute hydrocephalus
No	27	5.9 ± 0.9	−0.448 (−0.436–0.276)	0.655	19.1 ± 5.2	1.031 (−1.209–3.821)	0.305
Yes	69	6.0 ± 0.8			17.8 ± 5.7
Chronic hydrocephalus
No	72	6.0 ± 0.8	1.041 (−0.175–0.561)	0.300	18.3 ± 5.3	0.452 (−2.026–3.220)	0.652
Yes	24	5.9 ± 0.8			17.7 ± 6.6
Cerebral infarction
No	65	6.0 ± 0.8	0.761 (−0.211–0.472)	0.448	18.6 ± 5.3	1.045 (−1.146–3.690)	0.299
Yes	31	5.9 ± 0.8			17.3 ± 6.2

*Independent samples t-tests comparisons between groups. Higher FSS and MFS scores indicate higher severity and/or intensity of fatigue*.

There was no significant relationship between fatigue and neurological impairment as scored with NIHSS [FSS: *r*_(94)_ = 0.06, *p* = 0.583; MFS: *r*_(94)_ = 0.17, *p* = 0.094]. There was also no significant relationship between fatigue and clinical outcome as scored with mRS [FSS: *r*_(94)_ = −0.06, *p* = 0.578; MFS: *r*_(94)_ = 0.17, *p* = 0.093] where all mRS 0 patients [*n* = 5] scored ≥4 on FSS and 60.0% [*n* = 3] scored ≥10.5 on MFS.

Weight gain was significantly associated with mean FSS scores [*r*_(94)_ = 0.25, *p* = 0.016], but not with MFS sum scores [*r*_(94)_ = −0.16, *p* = 0.128]. Further, weight gain was associated with depressive symptoms (BDI-II) [*r*_(94)_ = 0.27, *p* = 0.009], but did not relate to anxiety symptoms (BAI) [*r*_(94)_ = 0.06, *p* = 0.572]. When adjusted for depressive symptoms, mean FSS scores was no longer associated with weight gain [*r*_adj.(93)_ = 0.15, *p* = 0.140].

### Fatigue and Cognitive Function

**Figure 4:**
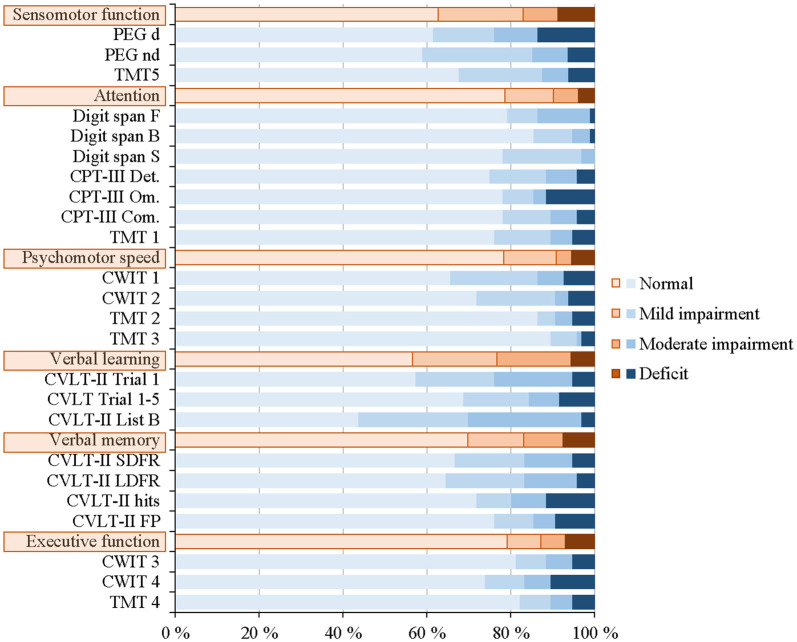
Distribution of normal, mild and moderate impairment, and deficit for the individual neuropsychological tests and the six cognitive domains for all 96 patients.

The neuropsychological test performances of the 96 patients are shown in Figure 4 (see also Supplementary Material: Neuropsychological test performance). As illustrated, the percentages of deficits were low and ranged from 3.9% to 8.7% within the six cognitive domains. The highest percentages of deficits were found for the domains “sensomotor function” [8.7%], “verbal memory” [7.6%], and “executive function” [6.9%].

Digit Span Forward (WAIS-IV) was negatively correlated to mean FSS scores [*r*_(94)_ = −0.21, *p* = 0.040, corrected: *p* = 0.960] and CVLT-II recognition Hits was negatively correlated with MFS sum scores [*r*_(94)_ = −0.22, *p* = 0.030, corrected: *p* = 0.720]. These associations were still significant when adjusted for mood disorders [*r*_adj.(93)_ = −0.25, *p* = 0.013, corrected: *p* = 0.312; *r*_adj.(93)_ = −0.26, *p* = 0.011, corrected: *p* = 0.264, respectively]. Grooved Pegboard dominant was negatively correlated with MFS sum scores [*r*_(94)_ = −0.20, *p* = 0.046, corrected: *p* = 1.0], but not when adjusted for mood disorders [*r*_adj.(93)_ = −0.19, *p* = 0.062]. However, none of the correlations remained statistically significant after Bonferroni correction. Hence, both fatigue scores were unrelated to the 24 neuropsychological test performance scores.

### Fatigue and Health-Related Quality of Life

Table 2 presents the results for the SF-36 subscales. Approximately half of the patients scored low (experienced problems) on the subscales role-physical, vitality, and social functioning.

We found significant negative correlations between both mean FSS and MFS sum scores and all SF-36 subscales; i.e., more fatigue correlated with poorer HRQoL (lower scores). All correlations reached *p* = ≤0.001 significance level except for the associations between fatigue scores and General health subscale which reached a *p* = <0.05 significance level [FSS: *r*_(94)_ = −0.32, *p* = 0.002; MFS: *r*_(94)_ = −0.20, *p* = 0.044]. After adjusting for mood disorders, mean FSS and MFS sum scores were still negatively correlated with all SF-36 subscales [*p* = <0.05] except for the association between MFS sum scores and General health subscale [*r*_adj.(93)_ = −0.07, *p* = 0.483].

### Fatigue and Return to Work

**Figure 5:**
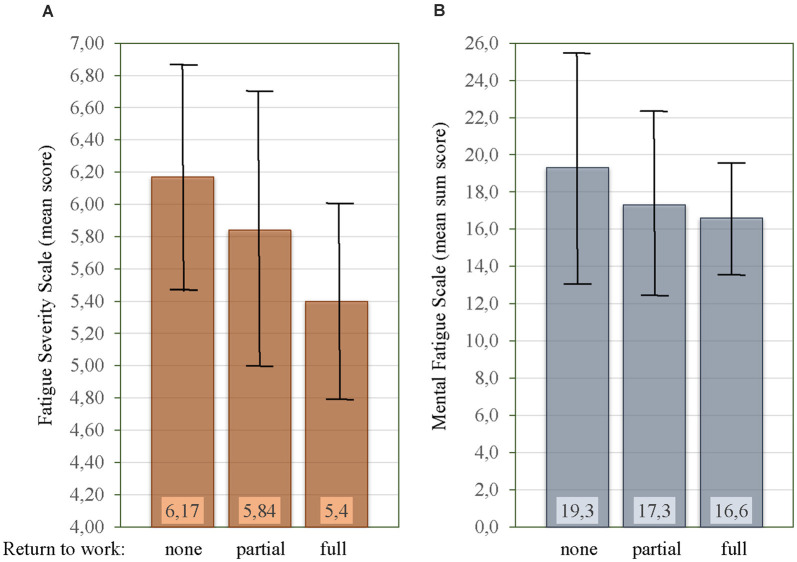
Relationship between mean Fatigue Severity Scale (FSS) score **(A)**, mean Mental Fatigue Scale (MFS) sum score **(B)**, and rate of return to work. Vertical bars on the histogram indicate ± 1 standard deviation.

Among the 78 patients that were employed at the time of hemorrhage; 43 [55.1%] had not returned to work (no RTW), 27 [34.6%] had partial RTW, and eight [10.3%] had full RTW. There was a significant difference between the mean FSS score for the different RTW categories (*F*_(2,75)_ = 4.17, *p* = 0.019, Figure 5A). The mean FSS score was significantly lower in patients with full RTW [5.40 ± 0.60] as compared to no RTW [6.17 ± 0.69, *p* = 0.005], but not when compared to partial RTW [5.84 ± 0.87, *p* = 0.085]. No significant difference in mean FSS score was seen between partial RTW and no RTW [*p* = 0.196]. Figure 5B shows the MFS sum score for the three RTW categories, which were not significantly different [*F*_(2,75)_ = 1.50, *p* = 0.231]. There was further no significant difference in depressive symptoms (BDI-II) scores [*F*_(2,75)_ = 1.65, *p* = 0.199] or anxiety symptoms (BAI) scores [*F*_(2,75)_ = 2.93, *p* = 0.060] for the different RTW categories.

## Discussion

Our patients experienced fatigue as being among their three most disabling symptoms and when characterizing their fatigue they emphasized the questionnaire items “low motivation,” “mental fatigue,” and “sensitivity to stress”. Fatigue due to exercise was the least bothersome aspect of fatigue and weight gain was associated with depressive symptoms rather than the severity of fatigue. Although there was a strong association between fatigue and mood disorders, especially for depression, the overlap was incomplete. Post-aSAH fatigue related to reduced HRQoL, and contributed to low rates of RTW.

### Post-aSAH Fatigue

Our itemized analysis favored high scores for questionnaire items that mostly are linked to a mental type of fatigue, whereas those linked to physical activity scored relatively low. Patients reported a disproportionately large drainage of mental energy after executing cognitive tasks or after engaging in a conversation with several people (MFS item 3). Motivation levels were low when fatigued (FSS item 1) and there was a reduced ability to cope with stress and manage tasks under time pressure (MFS item 8). These are typical features of mental fatigue, described as a dynamic process with fluctuation of mental energy levels. Buunk et al. (2018) found a frequency of mental fatigue of 48.4% and of physical fatigue of 38.5% in patients with aneurysmal and non-aneurysmal SAH, which indicates a stronger component of physical fatigue than our results suggest. The instrument used for measuring fatigue was, however, different as Buunk et al. (2018) used The Dutch Multifactor Fatigue Scale. Sörbo et al. (2019) investigated post-aSAH fatigue with the MFS questionnaire only and found 57% of patients scoring ≥10.5 points. They used no other questionnaires and their evaluation of physical fatigue was hence not beyond the means of the present study.

A relationship between pre- and post-stroke fatigue has been reported both in the acute (Lerdal et al., 2011) and chronic phase (Choi-Kwon et al., 2005; Lerdal et al., 2012). We did not find a significant difference between scores of fatigue in patients with or without a prior history of fatigue. To the best of our knowledge, no data regarding the relationship of pre- and-post-aSAH fatigue have previously been published. We interpret this result, in line with the other findings in the present study, to further support the notion that post-aSAH fatigue is primarily a result of pathological mechanisms caused by the hemorrhage itself.

### Fatigue and Mood Disorders

Although the prevalence of mood disorders after aSAH is high (Al-Khindi et al., 2010; Rinkel and Algra, 2011; Vetkas et al., 2013; Tang et al., 2020), few studies have examined the relationship between mood disorders and post-aSAH fatigue. Passier et al. (2011a) found higher fatigue scores 1 year after the ictus in patients scoring high on anxiety and depression than in those without such complaints 3 months after aSAH. Our results are in line with this; i.e., post-aSAH fatigue was strongly associated with depression. Furthermore, post-aSAH fatigue was to some degree associated with anxiety, but to a lesser extent than depression.

Even though post-aSAH fatigue and mood disorders were strongly associated, the overlap was incomplete since a majority of our patients did not have clinical depression or anxiety (65.6% and 81.2%, respectively). Stroke studies have demonstrated that fatigue may occur in the absence of depression (Ingles et al., 1999; van der Werf et al., 2001; Choi-Kwon et al., 2005). Our results suggest that this is also the case for fatigue and mood disorders after aSAH. Fatigue and mood disorders can exist independently, supporting the more commonly accepted notion that these two are distinct entities. Our itemized analysis of the fatigue questionnaires adds to the existing literature that the profile of fatigue characteristics is reported differently in those with post-aSAH fatigue and those with mood disorders. This latter subgroup reported more often that exercise brings on fatigue (FSS item 2), that fatigue causes frequent problems (FSS item 5), and that fatigue causes frequently problems with work, family, and social life (FSS item 9). Furthermore, those with mood disorders reported higher scores for sensitivity to stress (MFS item 8), emotional instability (MFS item 9), irritability (MFS item 10), and sensitivity to noise (MFS item 12). This subgroup may therefore need special attention during rehabilitation.

The temporal relationship between fatigue and mood disorders is not well understood. It is uncertain whether mood disorders after hemorrhage are influenced by personal factors, the aSAH itself, or by its consequences. We found no relationship between mood disorders and aSAH characteristics, which might suggest that mood disorders after aSAH are less likely to be a consequence of direct organic brain injury. However, patients with prior history of mood disorders, and especially depression, had higher scores on BDI-II and BAI than patients without this prior history. A suboptimal coping style could possibly serve as a predisposition for mood disturbance after aSAH (Noble et al., 2008) and therefore indirectly be related to post-aSAH fatigue.

### Fatigue and Demographic Data/aSAH Characteristics

Previous studies have failed to show significant relationships between post-aSAH fatigue and age, gender, education level, aneurysm localization, and treatment modality (Passier et al., 2011a; Western et al., 2020). We presently found higher mean FSS scores but similar MFS sum scores among those with low/intermediate as compared to high education level. Selection bias might be of importance as our aSAH population was on average highly educated, whereas the population in Passier et al. (2011a) had primarily low education. The higher mean FSS scores in our patients with endovascular aneurysm repair could be due to the larger fraction of patients with mood disorders in that group.

Sörbo et al. (2019) demonstrated a correlation between MFS score and functional outcome after aSAH as expressed with GOSE, where none of their fully recovered patients (GOSE 8) had an MFS sum score suggestive of mental fatigue (≥10.5). Presently, all five mRS 0 patients scored ≥4 on FSS and three of them scored ≥10.5 on MFS. The mRS may be less sensitive to certain functional impairments as compared to the GOSE. The mRS was presently scored by the clinician during the control visit. A score of mRS 0 may indicate that significant fatigue may be overlooked in a regular interview, especially in patients that have no neurological impairments. Due to the exclusion criteria of our study, we included patients that had very few neurological impairments, and hence one would not expect to find a clear relation between neurological status and fatigue.

Depressed individuals are at higher risk for developing obesity than non-depressed individuals (Blaine, 2008). This can explain that weight gain in our aSAH patients was related to depressive symptoms and not to fatigue. Weight gain was hence no surrogate marker for physical fatigue. Even though FSS item 2 “Exercise brings on my fatigue” was scored higher in those with mood disorders, it was the least prominent FSS item also in that subgroup.

### Fatigue and Cognitive Function

Numerous studies have documented cognitive sequelae after aSAH (Al-Khindi et al., 2010; Rinkel and Algra, 2011; Nordenmark et al., 2019b; Burke et al., 2020; Nussbaum et al., 2020). Despite increasing interest in using neuropsychological tests in fatigue research, a lack of correlation between fatigue and neuropsychological performance has been observed across a wide variety of clinical samples (DeLuca, 2005). This concurs with the present findings where few of our patients had cognitive deficits despite all of them suffering from fatigue. According to DeLuca (2005), the most consistent finding is for subjective fatigue to be more closely related to depression than with objective cognitive performance. This is also in line with our results.

Passier et al. (2011a) demonstrated that aSAH patients with cognitive impairments reported a higher level of fatigue than those without cognitive impairment. In their subgroup of patients with neither physical nor cognitive impairment, however, passive coping style and emotional problems were considered important predictors of fatigue. This could possibly also be so in the present study as our patient cohort of 96 patients closely resembled their subgroup of patients without physical and cognitive deficits. Assessment of coping style among our patients could hence have provided valuable information regarding fatigue.

### Fatigue and HRQoL/RTW

It is well-documented that reduced HRQoL is a common sequel after aSAH (Visser-Meily et al., 2009; Al-Khindi et al., 2010; Rinkel and Algra, 2011; Wong et al., 2011; Czapiga et al., 2013; Passier et al., 2013; Taufique et al., 2016). Visser-Meily et al. (2009) found post-aSAH fatigue to be strongly related to a decreased HRQoL. Our findings, where both high mean FSS scores and MFS sum scores were strongly associated with abnormal findings in all subscales of SF-36, concur with that. Even after adjusting for mood disorders, known to be related to reduced HRQoL (Visser-Meily et al., 2009), most of the subscales on SF-36 presently correlated with the post-aSAH fatigue scores.

Limitation in physical and social activities and low vitality were presently reported as the most reduced aspects of HRQoL. Despite their good neurological function, half of our patients still reported physical limitation as the most affected impairment. This was mainly due to their fatigue and not because they actually were physically impaired. Czapiga et al. (2013) also found this subscale to be most impaired in an aSAH population so that this could possibly reflect a physical component of fatigue. A limitation of social activities was also reported as one of the most affected aspects of quality of life. Drainage of mental energy and stress hyper-sensitivity poses a challenge for engagement in social activities. Social withdrawal may therefore be interpreted as a coping mechanism to reduce fatigue.

Despite high functional independence and low frequency of cognitive deficits, we found higher mean FSS scores to be related to a lower rate of RTW. This may be due to a partial overlap in measurement as FSS quantifies the impact of fatigue on daily life. Measuring fatigue with the MFS could not reproduce that clear relationship to RTW, but may perhaps have done so in a larger sample. Mood disorders could not explain our low rate of 10.3% of RTW. Several studies have reported that aSAH survivors have a surprisingly low rate of RTW although being physically capable of working (Hop et al., 2001; Wermer et al., 2007; Passier et al., 2011b; Wallmark et al., 2016; Nordenmark et al., 2019a). Even though the low rate of RTW after aSAH probably is a multifactorial problem, our study demonstrates that fatigue is an important factor to consider when planning occupational therapy. The consequence of the low rate of RTW is not only of economical and psychosocial importance for the relatively young aSAH population but also for society.

### Implications

Some have questioned if a so-called good outcome justifies the assumption that aSAH patients have no relevant neurobehavioral impairments (Hütter et al., 1999). Our findings support the notion of an invisible dysfunction after aSAH. All our patients were good outcome (mRS 0–2) where majority had no signs of cognitive deficit, nevertheless, they experienced a debilitating and long-lasting fatigue, sometimes in conjunction with emotional problems, with a major impact on perceived quality of life and ability to return to work. This apparent discrepancy is of importance in clinical practice. It is crucial that clinicians early in the recovery process can identify and acknowledge that fatigue is distinct from normal exhaustion and may be part of a long-lasting illness trajectory with a huge impact on social and occupational engagement. The aSAH survivor and their families are in need of information about the prevalence and typical characteristics of post-aSAH fatigue in order to have a better foundation for realistic expectations regarding recovery and coping. Acknowledging post-aSAH fatigue as a health problem can facilitate coping.

Since our results support the notion of fatigue and depression as distinct entities, it appears sensible to treat the latter with antidepressants. A relief of depressive symptoms has the potential to reduce symptom severity of fatigue although not being a cure of fatigue *per se*. Fatigue as the predominant contributor of the inability of RTW will, however, not be affected by antidepressant treatment. Since physical fatigue and impairment is not a dominant problem, principles of stroke rehabilitation with a stronger focus on physical training may not be helpful or even counterproductive in aSAH patients (Johansson and Rönnbäck, 2014).

Our findings also highlight the need for more research. The present study emphasizes the need for more knowledge on the typical features of fatigue after aSAH. A better understanding of which fatigue features to focus on and how to measure these aspects in a standardized manner could generate empirical evidence about the ossible underlying mechanism of fatigue and will therefore endorse the development of evidence-based treatment for fatigue. To this date, there is still insufficient evidence to support the use of any intervention to treat or prevent fatigue after stroke (Wu et al., 2015).

### Limitations

Our results have to be interpreted in light of the strict selection of participants based on the inclusion and exclusion criteria of the RCT they were recruited into. This resulted in all participants being good outcome (mRS 0–2) aSAH survivors without significant neurological or cognitive deficits. Furthermore, participation in our RCT, from where the present data were extracted, was comprehensive, many patients would have to travel over long distances to participate, and many patients, therefore, declined to participate. The RCT participants may thus have unknown common features affecting the profiles presently investigated. Nevertheless, since there were no significant differences between included and excluded patients with regard to gender, aneurysm location, and mode of aneurysm repair, and as we included patients over the entire specter of aSAH severity (even more in poor grade than among those excluded), the present findings should be representative for all patients with post-aSAH fatigue. It appears paradoxical that the deadliest type of intracerebral hemorrhage results in more neurologically intact survivors than ischemic stroke; it underlines, however, that the post-aSAH fatigue group is diverse from the post-stroke group that often struggles with neurological deficits. Selecting patients with post-aSAH fatigue and significant neurological impairment may have produced a different nature of fatigue in our itemized analysis. On the other hand, including only good outcome patients pinpoints the detrimental effects of fatigue after aSAH.

There are no validated instruments to assess fatigue after aSAH. Although the mental features of fatigue are not properly measured with FSS, it is still the most frequently used questionnaire for evaluating fatigue in stroke studies due to its high internal consistency (Nadarajah et al., 2017). The MFS is a new questionnaire for assessing mental fatigue after mild TBI, and its psychometric properties are not extensively studied in the aSAH population. On the other hand, there are strong similarities to the clinical picture in the chronic phase after mild TBI and aSAH, suggesting that the MFS questionnaire may also be a useful tool in the evaluation of post-aSAH fatigue. The MFS incorporates items addressing cognitive complaints like “Concentration difficulties” (item 5), “Memory problems” (item 6), and “Slowness of thinking” (item 7) which are not directly linked to fatigue. The score on these items will hence increase the MFS sum score, without a higher score necessarily reflecting more fatigue. It is noteworthy that our patients scored all of these three items higher than 1.0 on average even though the majority of their neuropsychological test performances were within the normal range. The individual perception of cognitive problems may hence also be an aspect of fatigue. Future studies of fatigue after aSAH should therefore employ and validate the use of questionnaires that address the multidimensionality of post-aSAH fatigue.

Also, the BDI-II and to some extent the BAI contain items that could be experienced by patients with post-aSAH fatigue but not necessarily the symptoms for which the measures were developed. Although we attempted to correct for this overlap by setting a conservative cut-off for the BDI-II and BAI, the potential for symptom overlap still exists.

### Conclusions

Good outcome patients with post-aSAH fatigue experienced their fatigue as being among the three most disabling symptoms and when characterizing their fatigue they emphasized the questionnaire items “low motivation,” “mental fatigue,” and “sensitivity to stress”. Fatigue due to exercise was the least bothersome aspect of fatigue and weight gain was associated with depressive symptoms rather than the severity of fatigue. Although there is a strong association between post-aSAH fatigue and mood disorders, especially depression, the overlap is incomplete and our findings support the notion that these symptoms are distinct entities. Post-aSAH fatigue often exists without significant neurological or cognitive impairments but relates significantly to reduced HRQoL and contributes to a low rate of return to work.

## Data Availability Statement

The datasets presented in this article are not readily available because it provides information about an unpublished RCT study. Requests to access the datasets should be directed to Elin Western, elin.western@gmail.com.

## Ethics Statement

The studies involving human participants were reviewed and approved by Regional ethics committee (reference: 2016/2214). The patients/participants provided their written informed consent to participate in this study.

## Author Contributions

EW, AS, and TN designed the research. EW, AS, WS, TK, and TN acquired the data. EW and AS analyzed the data. EW, AS, WS, and TN interpreted the results. EW drafted the manuscript. AS, WS, TK, and TN edited the manuscript. All authors contributed to the article and approved the submitted version.

## Conflict of Interest

The authors declare that the research was conducted in the absence of any commercial or financial relationships that could be construed as a potential conflict of interest.
