# Supplementary material for: Fatigue After Aneurysmal Subarachnoid Hemorrhage: Clinical Characteristics and Associated Factors in Patients With Good Outcome
Source: Front Behav Neurosci. 2021 May 12;15:633616. doi: 10.3389/fnbeh.2021.633616 (PMC8149596; doi:10.3389/fnbeh.2021.633616)
Supplement: Supplementary file 1 [file Table_1.DOCX]

Supplementary Material

# Supplementary Table

| Domain task | Normal | | Mild  impairment | | Moderate  impairment | | Deficit | |
| --- | --- | --- | --- | --- | --- | --- | --- | --- |
|  | *n* | % | *n* | % | *n* | % | *n* | % |
| Sensomotor function | | | | | | | | |
| Grooved Pegboard, dominant | 59 | 61.5 | 14 | 14.6 | 10 | 10.4 | 13 | 13.5 |
| Grooved Pegboard, non-dominant* | 56 | 58.9 | 25 | 26.3 | 8 | 8.4 | 6 | 6.3 |
| D-KEFS - Trail Making Test 5 | 65 | 67.7 | 19 | 19.8 | 6 | 6.3 | 6 | 6.3 |
| Attention | | | | | | | | |
| D-KEFS - Trail Making Test 1 | 73 | 76.0 | 13 | 13.5 | 5 | 5.2 | 5 | 5.2 |
| WAIS-IV - Digit span, forward | 76 | 79.2 | 7 | 7.3 | 12 | 12.5 | 1 | 1.0 |
| WAIS-IV - Digit span, backward | 82 | 85.4 | 9 | 9.4 | 4 | 4.2 | 1 | 1.0 |
| WAIS-IV - Digit span, sequence | 75 | 78.1 | 18 | 18.8 | 3 | 3.1 | 0 | 0.0 |
| CPT-III - Detectability | 72 | 75.0 | 13 | 13.5 | 7 | 7.3 | 4 | 4.2 |
| CPT-III - Omissions | 75 | 78.1 | 7 | 7.3 | 3 | 3.1 | 11 | 11.5 |
| CPT-III - Commissions | 75 | 78.1 | 11 | 11.5 | 6 | 6.3 | 4 | 4.2 |
| Psychomotor speed | | | | | | | | |
| D-KEFS – Color-Word Interference Test 1 | 63 | 65.6 | 20 | 20.8 | 6 | 6.3 | 7 | 7.3 |
| D-KEFS – Color-Word Interference Test 2 | 69 | 71.9 | 18 | 18.8 | 3 | 3.1 | 6 | 6.3 |
| D-KEFS - Trail Making Test 2 | 83 | 86.5 | 4 | 4.2 | 4 | 4.2 | 5 | 5.2 |
| D-KEFS - Trail Making Test 3 | 86 | 89.6 | 6 | 6.3 | 1 | 1.0 | 3 | 3.1 |
| Verbal learning | | | | | | | | |
| CVLT-II - Trial 1 | 55 | 57.3 | 18 | 18.8 | 18 | 18.8 | 5 | 5.2 |
| CVLT-II - Learning (Trial 1-5) | 66 | 68.8 | 15 | 15.6 | 7 | 7.3 | 8 | 8.3 |
| CVLT-II - List B | 42 | 43.8 | 25 | 26.0 | 26 | 27.1 | 3 | 3.1 |
| Verbal memory | | | | | | | | |
| CVLT-II - Short term memory | 64 | 66.7 | 16 | 16.7 | 11 | 11.5 | 5 | 5.2 |
| CVLT-II - Long term memory | 62 | 64.6 | 18 | 18.8 | 12 | 12.5 | 4 | 4.2 |
| CVLT-II - Recognition, hits | 69 | 71.9 | 8 | 8.3 | 8 | 8.3 | 11 | 11.5 |
| CVLT-II - Recognition, false positive | 73 | 76.0 | 9 | 9.4 | 5 | 5.2 | 9 | 9.4 |
| Executive function | | | | | | | | |
| D-KEFS – Color-Word Interference Test 3 | 78 | 81.3 | 7 | 7.3 | 6 | 6.3 | 5 | 5.2 |
| D-KEFS – Color-Word Interference Test 4 | 71 | 74.0 | 9 | 9.4 | 6 | 6.3 | 10 | 10.4 |
| D-KEFS - Trail Making Test 4 | 79 | 82.3 | 7 | 7.3 | 5 | 5.2 | 5 | 5.2 |

Abbreviations: D-KEFS = Delis-Kaplan Executive Function System; WAIS-IV = Wechsler Adult Intelligence Scale – fourth edition; CPT-III = Conners’ Continuous Performance - 3^rd^ edition; CVLT-II = California Verbal Learning Test – Second edition. *1 missing response.
